# DPP promotes odontogenic differentiation of DPSCs through NF-κB signaling

**DOI:** 10.1038/s41598-021-01359-3

**Published:** 2021-11-11

**Authors:** Yinghua Chen, Adrienn Pethö, Amudha Ganapathy, Anne George

**Affiliations:** Brodie Tooth Development Genetics and Regenerative Medicine Research Laboratory, Department of Oral Biology, University of Illinois at Chicago, Chicago, IL 60612 USA

**Keywords:** Organogenesis, Stem cells, Transdifferentiation, Cell growth, Cell signalling, Transcription

## Abstract

Dentin phosphophoryn synthesized and processed predominantly by the odontoblasts, functions as both structural and signaling protein. Mechanistic studies revealed that DPP stimulation of DPSCs positively impacted the differentiation of DPSCs into functional odontoblasts. Results show that NF-κB signaling and transcriptional activation of genes involved in odontoblast differentiation were influenced by DPP signaling. Specifically, RelA/p65 subunit of NF-κB was identified as being responsible for the initiation of the differentiation cascade. Confocal imaging demonstrated the nuclear translocation of p65 with DPP stimulation. Moreover, direct binding of nuclear NF-κB p65 subunit to the promoter elements of Runx2, Osx, OCN, MMP1, MMP3, BMP4 and PTX3 were identified by ChIP analysis. Pharmacological inhibition of the NF-κB pathway using TPCA-1, a selective inhibitor of IKK-2 and JSH-23, an inhibitor that prevents nuclear translocation and DNA binding of p65 showed impairment in the differentiation process. Functional studies using Alizarin-Red staining showed robust mineral deposits with DPP stimulation and sparse deposition with defective odontoblast differentiation in the presence of inhibitors. In vivo expression of NF-κB targets such as OSX, OCN, PTX3 and p65 in odontoblasts and dental pulp cells from DSPP null mouse was lower when compared with the wild-type. Overall, the results suggest an important role for DPP-mediated NF-κB activation in the transcriptional regulation of early odontogenic markers that promote differentiation of DPSCs.

## Introduction

Biomineralization, the generation of mineralized tissues is a common process by which living organisms produce hard tissues composed of biominerals. During this process, the organism defines the shape and structure of the mineralized tissue using complex regulatory signaling networks to synthesize a well-defined extracellular matrix that has both structural and signaling function^1,2^. The composition of the extracellular matrix in calcified tissues is unique^3,4^. It not only functions as a scaffold for cell survival but also serves to provide vital communications to instruct cells to synthesize and remove various components of the matrix in a temporal sequence. Cells control the process by synthesizing an organic structural matrix which by itself will not mineralize^5^. In bone and dentin, these matrices are the collagen fibril lattice. The cells then secrete matrix interactive proteins into well-defined positions in the self-assembled fibrillary matrix^6^. This process creates an interactive structural matrix, which then specifically attracts and localizes mineral ions, also secreted by the cells into the extracellular matrix. This process results in an instructive matrix that is conducive for mineral deposition and growth.

Dentin phosphophoryn (DPP) is a matrix interactive noncollagenous protein predominantly found in the dentin matrix^7,8^. DPP was initially thought to be a structural protein that functions to bind calcium and specify the placement of the mineral crystal with respect to the collagenous matrix^9,10^. Calcium binding property of DPP was attributed to its high negative charge contributed by amino acid residues such as aspartic acid and serines which make up about three-fourths of the total amino acids^11^. Further, phosphorylation of the serine residues increases the acidic nature of this protein^12^. Besides its structural function in the matrix, DPP functions in cell signaling events. The signaling function of DPP was demonstrated during embryonic development of the kidneys particularly facilitating epithelial-mesenchymal interactions in meristic tissues^13^. We have also demonstrated that the RGD domain in DPP is functional and signals through cell surface integrins^9,14^. Further, we have demonstrated that treatment of undifferentiated mesenchymal cells can stimulate the release of intracellular Ca^2+^ and promote osteogenic gene expression. However, little is known regarding the transcription factors and signaling pathways by which DPP mediates the commitment and terminal differentiation of dental pulp stem cells into odontoblasts.

Dental caries results in an inflammatory environment in the dental–pulp complex. It is now known that inflammatory process is a prerequisite for healing and regeneration in the dentin–pulp complex^15^. Recently, we have demonstrated that DPP stimulation can activate NF-κB^9^. The NF-κB family comprises of 5 closely related members: p65/Rel A, c-Rel, NF-κB1/p50. This transcription factor when inactive resides in the cytoplasm as heterotrimeric complex comprised of p50/p52, p65 and Ikappa B (IκB) inhibitor which masks the nuclear localization sequence of NF-κB and prevents its nuclear localization and subsequent DNA binding^16^. Upon phosphorylation of IκB by upstream kinases the complex is disrupted. Dissociated p-IκB is ubiquitinated and degraded, while the rest of the heterodimeric complexes of p50/p65 and p52/p65 translocate to the nucleus to perform transcriptional functions^17^.

To examine the process of odontogenic differentiation, dental pulp stem cells (DPSCs) were used in this study as they have demonstrated great potential to be used in regenerative medicine for dental-related diseases^18^. DPSCs are adult multipotent stem cells derived from both neural crest and mesenchyme and have the potential to differentiate into multiple cell lineages^18^. During tooth development, multiple signaling pathways such as BMP, Wnt, FGF, SHH are known to play significant role, however, the role of NF-κB signaling in tooth development, particularly, with respect to matrix mineralization, are not fully understood^19^. Loss-of-function studies have identified a role for NF-κB signaling in the specification of cusp formation in molar tooth development^20^. Mice overexpressing *Iĸĸβ* an essential component of NF-κβ signaling driven by K5 promoter, showed supernumerary incisors, which initiated from embryonic epithelium, suggesting that higher activity of NF-κB signaling activates uncontrolled odontogenic activity in embryonic incisor epithelium^19^.

NF-κB activation occurs in response to various stimuli. In this study, we report that DPP activates NF-ĸB signaling in DPSCs to promote odontogenic lineage differentiation. Interestingly, terminal differentiation of DPSCs and matrix mineralization were specific to NF-κB subunit p65, an activity that might be important for odontoblast differentiation and proper synthesis of an ECM responsible for mineralization.

## Results

### DPP activates NF-κB signaling pathway in DPSCs

**Figure 1:**
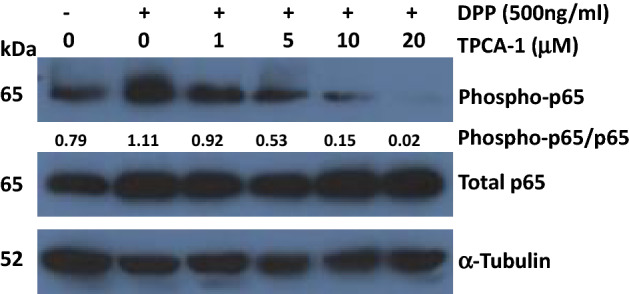
Activation of NF-κB subunit p65 by DPP. DPSCs were treated with DPP in the presence and absence of the NF-κB inhibitor TPCA-1. Varying concentrations of TPCA-1 were used as indicated. Total and phosphorylated levels of NF-κB subunit p65 were examined by Western blot. Note higher concentrations of TPCA-1 blocked activation of p65. Tubulin was used as a loading control. The relative density ratio of phospho p65 to total p65 protein bands normalized to tubulin are shown.

We first examined if stimulation of DPSCs with rDPP activated NF-κB signaling through phosphorylation of the p65 subunit of NF-κB (Ser-536) (Fig. 1). Treatment of DPSCs with TPCA-1 a selective inhibitor of IKK-2 showed a dose-dependent decrease in the levels of p65 phosphorylation (Fig. 1). Complete loss of phosphorylation was observed with the highest dose of the inhibitor. Thus, DPP stimuli specifically activates RelA/p65 subunit of NF-κB.

**Figure 2:**
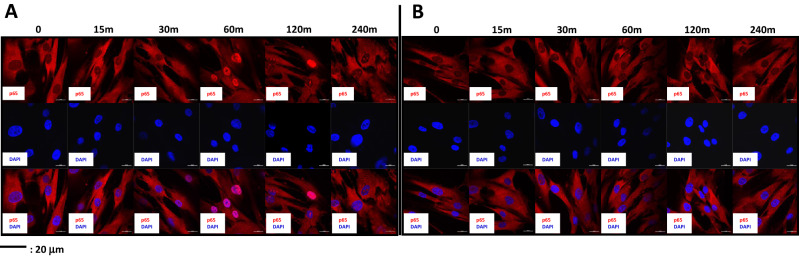
Confocal microscopy imaging to demonstrate the nuclear translocation of p65 by DPP stimulation. (**A**) DPSCs were stimulated with DPP (500 ng/ml) for indicated times and cellular localization of p65 (Red) was assessed by confocal microscopy. DAPI (blue) depicts the nucleus. Scale bar: 20 µm (**B**) Cellular localization of p65 was determined after treating DPSCs with the NF-κB inhibitor TPCA-1 (10 µM) for 1 h and then stimulated with DPP at the indicated times. Note absence of nuclear localization of p65 with TPCA-1 treatment.

Phosphorylation of Ser536 in the cytosol by IKK promotes its nuclear translocation^21^. Therefore, we monitored the translocation of p65 to the nucleus by immunocytochemistry. In the absence of DPP stimulation, NF-κB was predominantly localized in the cytoplasm. With DPP stimulation, p65 rapidly accumulated in the nucleus within 30 min with maximum localization at 60 min (Fig. 2A). After 240 min p65 was no longer detected in the nucleus. Thus, NF-κB activation by DPP is rapid and transient. Specificity of phosphorylation of NF-κB by IKK and its subsequent nuclear translocation was demonstrated by pretreatment with IKK-kinase inhibitor TPCA-1. Results in Fig. 2B showed that TPCA-1 completely blocked nuclear translocation even with DPP stimulation.

### DPP stimulates cell growth by activating-NF-κB

**Figure 3:**
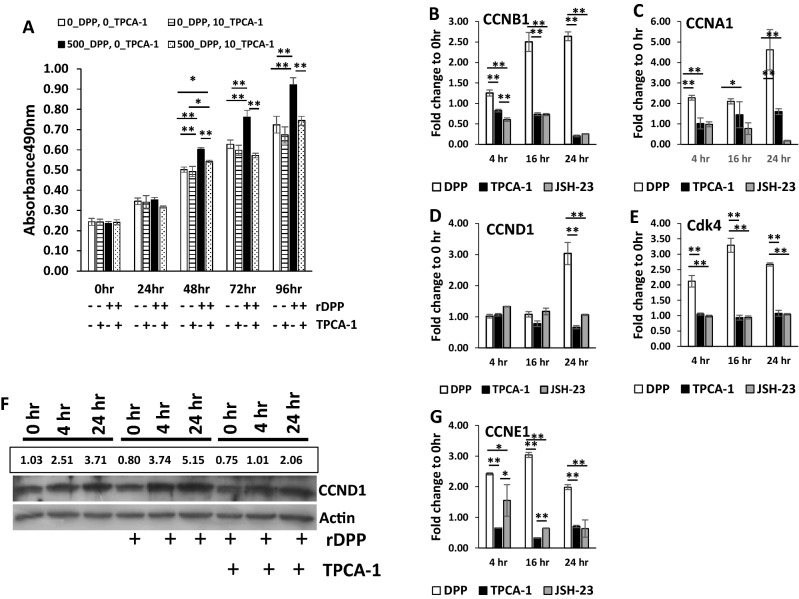
DPP promotes cellular proliferation by NF-κB activation. (**A**) DPSCs were cultured were treated with (closed bar) or without TPCA-1 (open bar) for 60 min and stimulated with 500 ng DPP and cultured under growth conditions for 0–96 h. (**B**) DPSCs were treated with or without TPCA-1 for 60 min and then stimulated with DPP for 0, 4 and 24 h. Western blot analysis was performed to assess the expression levels of cyclin D1. (**C**) DPSCs were treated with or without NF-κB pathway inhibitors TPCA-1 or JSH-23 for 60 min and then stimulated with DPP at the indicated time points. Expression of cyclin B1 levels were assessed by RT-PCR. Fold change was obtained relative to 0 h. (**D**) DPSCs were treated with or without NF-κB pathway inhibitors TPCA-1 or JSH-23 for 60 min and then stimulated with DPP at the indicated time points. Expression of cyclin D1 levels were assessed by RT-PCR. Fold change was obtained relative to 0 h. (**E**) DPSCs were treated with or without NF-κB pathway inhibitors TPCA-1 or JSH-23 for 60 min and then stimulated with DPP at the indicated time points. Expression of cyclin E1 levels were assessed by RT-PCR. Fold change was obtained relative to 0 h. (**F**) DPSCs were treated with or without NF-κB pathway inhibitors TPCA-1 or JSH-23 for 60 min and then stimulated with DPP at the indicated time points. Expression of cyclin A1 levels were assessed by RT-PCR. Fold change was obtained relative to 0 h. (**G**) DPSCs were treated with or without NF-κB pathway inhibitors TPCA-1 or JSH-23 for 60 min and then stimulated with DPP at the indicated time points. Expression of Cdk4 levels were assessed by RT-PCR. Fold change was obtained relative to 0 h.

The effect of DPP on cell growth were monitored by an MTS assay. Under growth conditions, DPSCs treated with DPP were significantly higher when compared with the unstimulated cells (Fig. 3A). Lower cell growth was observed in the presence of NF-κB inhibitor TPCA-1. We next examined if cellular growth is mediated by cyclins. Results in Fig. 3B,C show that Cyclin B1 and A1 were upregulated with DPP stimulation up to 24 h and expression levels were significantly attenuated in the presence of TPCA-1 and JSH-23 along with DPP stimulation. Interestingly, cyclin D1 showed maximum expression at 24 h, while Cdk4 showed higher expression at 16 h (Fig. 3D–F). Moreover, gene expression analysis of cyclin E1 was upregulated with DPP treatment at 4 and 16 h and lower with the addition of NF-κB inhibitors, TPCA-1 and JSH23 (Fig. 3G).

### DPP stimulation promotes odontogenic differentiation of DPSCs by activation of p65

**Figure 4:**
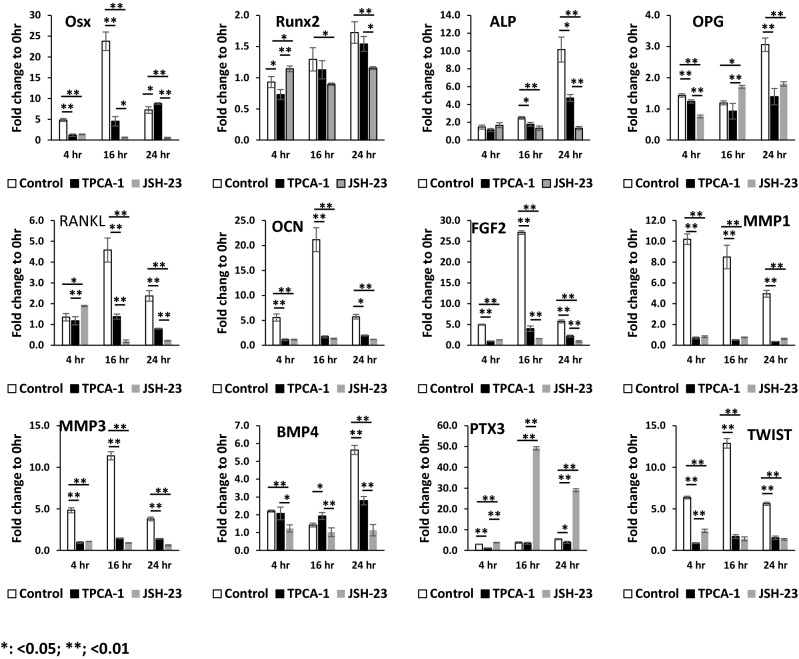
Effect of DPP-mediated NF-κB activation on the odontogenic differentiation of DPSCs. DPSCs were cultured under growth conditions and were treated with (open bars) or without TPCA-1 (closed bars) or JSH-23 for 60 min and stimulated with 500 ng DPP and cultured under growth conditions for 0–96 h. Total RNA was isolated and quantitative RT-PCR analysis performed. Fold changes were obtained relative to 0 h. Expression levels of early odontogenic markers such as; Osx, Runx2, Alp, OPG, RANKL, OCN, FGF2, MMP1, MMP3, BMP4, PTX3 and TWIST increased with DPP-mediated NF-κB signaling. Specificity of NF-κB signaling was determined by gene expression analysis in the presence of NF-κB signaling pathway inhibitors, TPCA-1 and JSH-23. Data are means ± SD of triplicates. Means and SDs of fold changes to 0 h are shown. Significant difference *p < 0.05; **p < 0.01.

We next examined the impact of nuclear p65 on odontogenic gene transcription in DPSCs by quantitative RT-PCR. Results in Fig. 4 show gradual increase in expression levels of early odontogenic genes such as Runx2, ALP, OPG, BMP4 and PTX3 from 4 to 24 h, while MMP1 expression was highest at 4 h and reduced levels at 24 h. Other genes that were examined such as FGF2, Osterix, RANKL, OCN, MMP3 and TWIST1 had higher expression levels at 16 h. Interestingly, pretreatment of DPSCs with the inhibitors TPCA-1 and JSH-23 effectively blocked the transcriptional activation of these genes, demonstrating the role of DPP-mediated p65 activation in the transcriptional regulation of several “early” genes for odontogenic differentiation (Fig. 4).

### DPP-mediated p65 activation promoted terminal differentiation of DPSC’s

**Figure 5:**
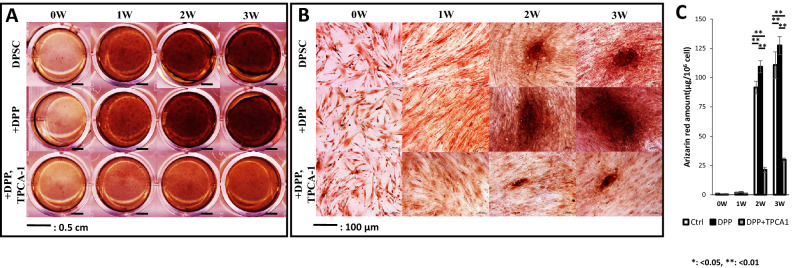
DPP stimulation promotes matrix mineralization by activation of NF-κB signaling. (**A**) DPSCs stimulated with 500 ng DPP in the presence and absence of the inhibitor TPCA-1 were cultured under differentiation conditions over a period of 0,7,14 and 21 days and mineralized nodules containing calcium were visualized using Alizarin Red staining. (**B**) Higher magnification of (A) show calcium deposits in the mineralization cultures at various time points. (**C**) Quantitative measurement of calcium deposition was determined by measuring the absorbance of the eluted Alizarin Red stain at 562 nm on a multiplate reader using a standard calcium curve. Statistically significant differences are indicated at 2 and 3 week *p < 0.05, **p < 0.01.

DPSC cells were subjected to differentiation under mineralization conditions with or without DPP, and deposition of calcified matrix was visualized by Alizarin-Red stain. Imaging of the overall Alizarin-Red stained culture dishes with DPP treatment showed strong staining progressively increasing with differentiation and calcified matrix formation (Fig. 5A). Light microscopic images showed larger mineralized nodules with deeper color with DPP treatment at 2 and 3 weeks (Fig. 5B). These effects were abrogated in the presence of TPCA-1 Quantification analysis of the extracted dye revealed higher amount of calcium deposited in DPP treated samples at each time point (Fig. 5C). Interestingly, mineralized nodule assay in the presence of TPCA-1, exhibited matrix containing smaller mineralized nodules with lower calcium content.

**Figure 6:**
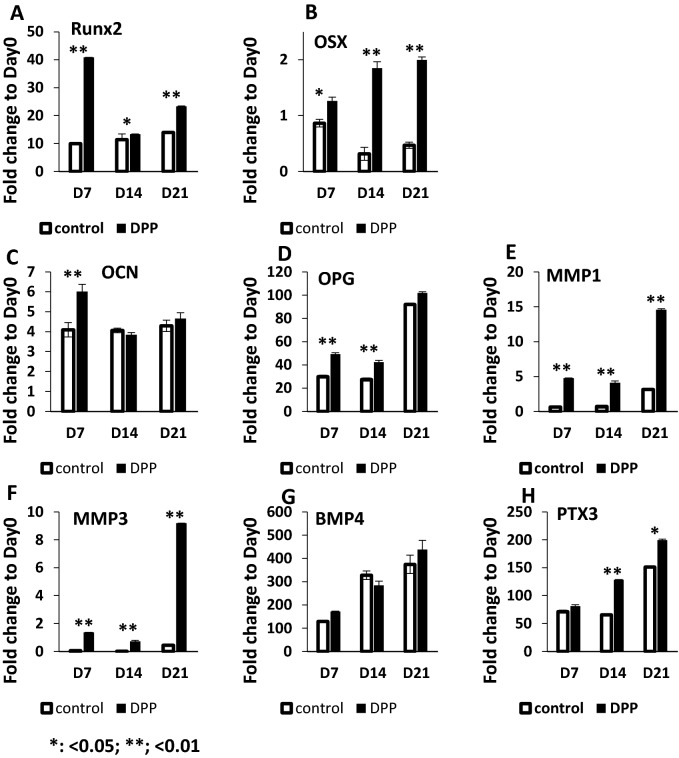
Expression of odontogenic markers during differentiation of DPSCs with DPP stimulation. Differentiation of DPSCs, with DPP stimulation were assessed by growing under osteogenic differentiation conditions over a period of 0,7,14 and 21 days. Expression of Osx, OPG, MMP1, MMP3, BMP4 and PTX3 showed an increase in fold change from 0–21 days. Expression levels of Runx2 and OCN peaked at 7 days. GAPDH expression was used as an internal control. Gene expression fold changes calculated as the relative ratios to day 0 and means and SDs of changes and comparisons are shown. *p < 0.05, **p < 0.01.

Changes in the expression levels of matrix genes during the terminal differentiation process were assayed by real time PCR. Osteogenic gene expression during the terminal differentiation process (Fig. 6) show high expression levels of Runx2 at day 7 and progressive increase in expression of Osterix up to 21 days (A and B). Runx2 and osterix are two essential transcription factors for odontoblast differentiation. Upregulation of osteocalcin an early matrix protein was observed at 7 days and decreased to control levels at 14 and 21 (C). Increase in OPG (osteoprotegerin) at 7 and 14 days was interesting as OPG has been recently implicated in reducing osteoclastic resorptive activity (D). High expression levels of MMP1 and MMP3 were observed throughout the differentiation process indicating a role in matrix remodeling (E and F). Increase in the expression levels of PTX3 with differentiation was significant (H). No significant changes were observed with BMP4 expression (G).

### DPP regulates promoter binding of NF-κB p65 subunit for the transcription of selected odontogenic genes

**Figure 7:**
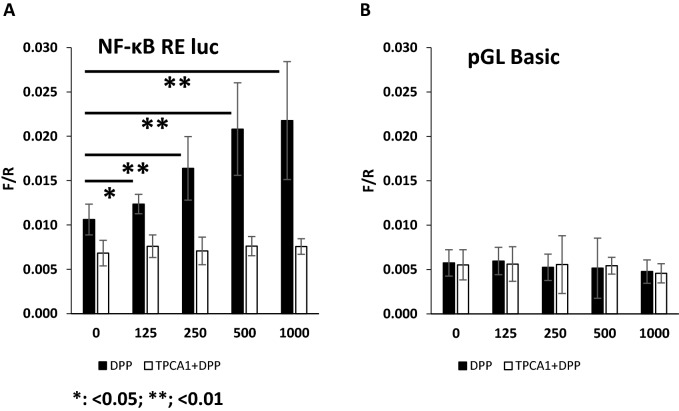
Activation NF-κB promoter with DPP stimulation. The NF-κB RE luciferase reporter (**A**) or the promoter less luciferase activity assay was performed using DPSCs stimulated with DPP in the presence or absence of p65 inhibitor TPCA-1. Luciferase activity of increased with DPP stimulation from 0–500 ng/ml (**A**). Activity was abrogated when cells were treated with TPCA-1 and then stimulated with DPP. Control pGL basic vector showed no activity when stimulated with DPP (**B**). The data are presented as means of ratio of firefly luciferase (F) to renilla luciferase (R), bars show mean ± SD. *p < 0.05, **p < 0.01.

We then assessed if DPSCs transiently transfected with an NF-κB luciferase reporter construct was activated by DPP. Results in Fig. 7A show that the luciferase promoter activity was gradually increased with addition of DPP at concentrations up to 500 ng/ml and then plateaued at 1000 ng/ml, while consistent low/basal level promoter activity was observed with pretreatment with the p65 inhibitor TPCA-1 along with DPP stimulation. PGL3-Basic luciferase vector that lacks eukaryotic promoter and enhancer domain, did not demonstrate change in activity irrespective of addition of DPP or TPCA-1 and DPP (Fig. 7B). This suggests that activated p65 traffics to the nucleus and binds to NF-κB sites in the promoter region of NF-κB luciferase, inducing its expression.

**Figure 8:**
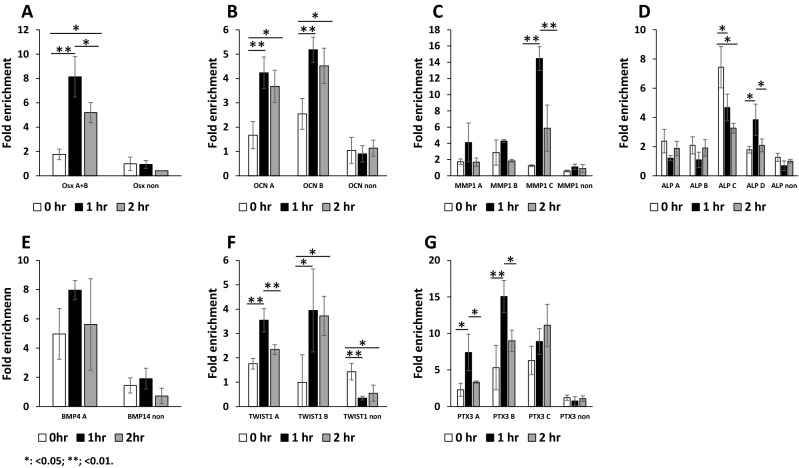
DPP-mediated NF-κB p65 activation directly associates with the odontogenic differentiation target gene promoter sequence containing NF-κB response elements in vivo. Sheared chromatin from DPSCs treated with or without DPP were used to perform ChIP assays. Cross-linked protein–DNA complexes were incubated with an anti-p65 antibody (closed bars) or control IgG antibody (open bars) on immunoprecipitated DNA was analyzed by qPCR using primers comprising the NF-κB sites on Osx, OCN, MMP-1, ALP, BMP4, TWIST1 and PTX3 promoters. Primer sequences are supplied in the supplementary section. The κB binding sites are designated from A–D in each of these promoters. A pair of random primers were used to demonstrate the specificity of binding. Results are expressed as percent of input DNA compared to IgG. Statistical significance are indicated as mean ± SD; *p < 0.05, **p < 0.01.

We next examined if the downstream NF-κB p65 target genes that were activated by DPP stimulation contained putative NF-κB binding sites. Bioinformatics was utilized to identify DNA elements with high homology to the p65 consensus sequence in several genes whose expressions were activated by DPP and blocked by the TPCA-1 inhibitor as shown in Fig. 4. The promoter region (2000 bp 5′-UTR, upstream from the transcription start site) were extracted from homo sapiens genome assembly GRCh38 using ENSEMBL genomic browser (https://uswest.ensembl.org/index.html) and the DNA fragments were searched against transcription factor binding sites using TFSEARCH (ver. 1.3) (http://diyhpl.us/~bryan/irc/protocol-online/protocol-cache/TFSEARCH.html). Genes that contained NF-κB binding sites were used in the ChIP assay. The ChIP assay were conducted as described in the “Materials and methods” section and the amount of the p65 bound DNA fragments were measured as percentages to total input DNA subjected to immunoprecipitation followed by qPCR. Upon DPP treatment, binding of p65 components of NF-κB to the putative binding sites in the promoter elements of several matrix genes such as OSX, OCN, MMP1, ALP, BMP4, TWIST1, and PTX3 showed distinct enrichment over the input chromatin compared to that of an IgG negative control antibody. No chromatin enrichment was observed with primers from adjacent random regions (Fig. 8). For the OSX promoter, the NF-κB binding chromatin enrichment upon DPP treatment peaked at 1 h, and then reduced but was still higher than without DPP stimulation (Fig. 7A). OCN DNA element A showed chromatin enrichment at 1 h and stayed high at 2 h, while element B peaked at 1 h (Fig. 8B). The MMP1 element C showed strong response to the DPP treatment at 1 h (Fig. 8C). With alkaline phosphatase, A and B elements p65 binding with DPP treatment showed slightly lower binding; while C element had consistent low binding upon DPP treatment; only enrichment of D element peaked at 1 h rDPP treatment (Fig. 8D). In the case of BMP4, A binding site was high at 0 h, and DPP stimulation at 1 h increased chromatin enrichment (Fig. 8E). DNA binding activity of TWIST1 A + B and C were increased upon DPP treatment, particularly at 1 h (Fig. 8F). Chromatin enrichment was observed with the three NF-κB DNA elements (A, B and C) in PTX3 (Fig. 8G).

### In vivo expression of downstream targets of NF-κB in *DSPP*-null mice

**Figure 9:**
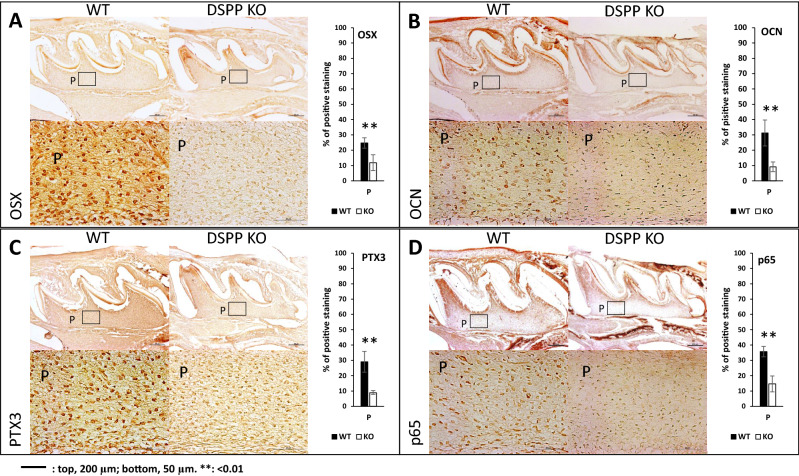
Expression of NF-κB targets in PN day 5 DSPP-KO and WT mice in the dentin-pulp complex. Expression of Osx (**A**), OCN (**B**), PTX3 (**C**) and p65 (**D**) in WT and DSPP KO are shown. Boxes marked have been magnified and the corresponding images showing staining in the pulp are shown in the bottom panel. Quantification of the immunohistochemical positive signals in the pulp are represented as bar graphs. Statistical significance **p < 0.01. *P* pulp.

We have previously shown that *DSPP-*KO mouse exhibit craniofacial anomalies particularly related to impaired mineralization of bone and teeth. Therefore, we examined the expression of three downstream targets of NF-kb signaling, namely osterix, OCN, PTX3 and the NF-κB subunit p65 using IHC. Results in Fig. 9 show higher expression levels of Osx, OCN, PTX3 and p65 in the dental pulp cells while lower expression levels were observed in DSPP-KO tissues confirming the requirement of DPP in odontogenic differentiation and matrix mineralization by activating NF-κB signaling. Quantification of the expression levels show significantly lower expression levels of these odontogenic markers in DSPP KO mice when compared to wild type (Fig. 9, bar graphs).

## Discussion

Dental pulp-derived stem cells are a promising source of adult stem cells which are currently being utilized for various regenerative applications due to their ability to self-renew and possess multilineage differentiation properties^22^. Dental pulp is an unmineralized connective tissue that is highly vascularized and innervated tissue and contains dental pulp stem cells, fibroblasts, endothelial, neurogenic and other specialized cell types such as odontoblasts at the periphery. The odontoblasts synthesize the dentin organic matrix, which comprises predominantly of collagenous matrix and noncollagenous proteins such as DPP^22^. During the progression of dental caries, dentin is degraded by enzymes and acids synthesized by the oral bacteria, causing the release of bioactive molecules which can promote the odontogenic differentiation of DPSCs^23^. One such predominant noncollagenous protein present in both the physiological and carious dentin matrix is dentin phosphophoryn^7,24^. Understanding the signaling pathway activated by DPP to promote the differentiation of DPSCs into odontogenic cells is necessary to device therapeutics for dentin repair and regeneration.

Transcription factor nuclear factor kappa B (NF-κB) regulates cellular responses to a wide variety of environmental cues. NF-κB activation is usually a rapid and transient response to a wide range of stimuli. In response to these stimuli, NF-κB proteins accumulate in the nucleus, bind to specific sequences in the genome and thus modulate gene transcription^21^. In this study, we demonstrate that DPP activates NF-κB and thereby initiate a signaling cascade which promotes the odontogenic differentiation of DPSCs. This is the first report to demonstrate that NF-κB is functionally activated by dentin phosphophoryn in DPSCs.

The Nuclear factor kappa B signaling system is a highly dynamic protein interaction network and the 5 subunits form 15 different combinations of homo or heterodimers. The most abundant form of NF-κB activated by the canonical pathway is the NF-κB p65^25^. The p65 monomer possess the transactivation domain (TAD) necessary for transcriptional activity^26^. Phosphorylation of serines in the TAD by CDK6 is necessary for binding to specific promoter sequences resulting in transactivation of genes and mediates context-dependent functional responses^21^. The NF-κB dimer p50/p65 is retained in the cytoplasm in an inactive form through its association with IκB. In the presence of a stimuli, a signaling cascade is activated leading to the phosphorylation of serine residues in IĸB resulting in ubiquitination and proteasomal degradation. This favors the release of NF-κB from its inhibitor and subsequently translocate to the nucleus, where it binds to ĸB consensus sequence encoded within its target gene and initiates transcription^27^. Canonical NF-κB signaling is normally a rapid and transient response to a wide range of stimuli.

The phosphorylation of IκB is a critical regulatory step in IκB degradation and subsequent NF-κB activation. The IKK kinase complex consists of two enzymatically active kinases, IKK-1 and IKK-2 and a regulatory subunit NEMO. However, the enzymes responsible for the ubiquitination of phosphorylated IκB are constitutively active. A plethora of NF-κB inhibitors have been developed which target different portions of the regulatory circuit^28^. Two small molecule inhibitors were used in this study to ensure the specificity of DPP-mediated NF-κB activation. TPCA-1 is an upstream selective small molecule inhibitor of NF-κB activity and it functions via attenuation of IKK-2 mediated phosphorylation of IĸB^27^. IĸB degradation unmasks the nuclear localization signal of NF-κB, allowing the transcription factor to translocate to the nucleus. JSH-23 the second inhibitor used in this study is a downstream inhibitor of NF-κB activity as it inhibits the DNA binding activity and nuclear translocation of NF-κB p65^16^. In this study, both inhibitors demonstrated attenuation of p65 activation and subsequent downregulation of odontogenic differentiation.

The NF-κB signaling play crucial roles in many physiological processes such as regulating organogenesis, stem cell regulations, cell proliferation and in several pathological processes such as immune and inflammatory responses^19^. The roles of NF-κB signaling in tooth development is not fully explored. In a recent study, overactivation of the canonical NF-κB pathway in ameloblasts resulted in abnormal enamel formation of the mouse molars. This was attributed to the compromised degradation of enamel protein at the maturation stage. In another study, overexpression of *Ikkß* under the keratin 5 promoter which is expressed in oral/dental epithelium induced enhanced odontogenic activity resulting in lingual incisor formation. Interestingly, the *K5-Ikkß* mice showed supernumerary incisors caused by up-regulation of canonical NF-κB signaling. Suppressing NF-κB activity failed to show any lingual incisors thus demonstrating that lingual incisor formation is dependent on increased canonical NF-κB signaling.

Dentin sialophosphoprotein (DSPP) is a precursor protein that is expressed by the connective tissues of the craniofacial skeleton, namely, dental pulp, bone and dentin^29,30^. After synthesis, DSPP is proteolytically cleaved into amino DSP and carboxyl-terminal DPP fragments^30,31^ . We have previously demonstrated that the DSPP gene contains an internal ribosome entry site that directs the synthesis of DPP^22^. Further, we also provide evidence that DPP is transported to the extracellular matrix through extracellular vesicles called exosomes^22^. Gene ablation studies show severe dental defects in *DSPP*-null mice^32^. Interestingly, transgenic expression of DPP partially rescued the dentin defects of *DSPP*-null mice^33^. In this study, we have observed reduced expression levels of NF-κB p65 in the dentin-pulp complex of day 5 *DSPP*-null mice. This indicates that the absence of DPP protein in the *DSPP*-null mice results in low levels of NF-κB that attenuates downstream signaling events. Other NF-κB targets that were downregulated in the mouse model were OCN, PTX3 and OSX. OCN and OSX are “early-genes” required for odontogenic differentiation while pentraxins (PTX3) which belong to a superfamily of multimeric proteins have been recently identified as a modulator of cell migration and odontogenic differentiation of DPSCs^34,35^.

In the present study, DPP stimulation promotes the odontogenic differentiation cascade of DPSCS. It is possible that DPP stimulates an inflammatory like response to activate p65 signaling and promote terminal differentiation of DPSCs. The early NF-κB downstream transcripts activated by DPP such as Runx2, Osx, PTX3, FGF2, BMP4, Twist1 and OCN might be responsible for the initiation of paracrine signaling to promote terminal differentiation markers. Down regulation of NF-κB activity also attenuated the gene expression of odontoblastic cell markers. Thus, the “early” odontogenic phenotype is regulated in DPSCs stimulated with DPP by activation of NF-κB signaling. Similar requirement of p65 activation was reported for endochondral ossification process^36,37^. Without external addition of chondrogenic growth factors, chondrocyte specific marker genes like Col2A1, Col10A1, Sox9 and aggrecan were observed in in vitro, ex-vivo and in-vivo endochondral models. This was attributed to the intensity and timing of activation of NF-κB/p65 signaling.

Nuclear translocation of p65 and binding to the respective promoter elements are responsible for transcriptional responses^38^. p65 subunit contains transactivation domains and contributes to the transcriptional activity of NF-κB^17,28^. Specifically, ChIP analysis confirmed p65 recruitment and binding to specific ĸB binding sites of the promoters of several NF-κB target genes. DPP mediated p65 activation was responsible for the regulation of early odontogenic transcription factors such as Runx2, Osterix, Twist1 and growth factors such as BMP4 that have been implicated in the differentiation of DPSCs to terminally differentiated odontoblasts. Pharmacological inhibitors that abrogated p65 activation downregulated gene expression. This suggests that transcriptional regulation of these odontogenic differentiation markers is facilitated by direct binding of p65 to multiple sites within the promoter region. However, we cannot exclude the role of other transcription factors that are required to function in a synergistic manner with NF-κB to obtain robust transcriptional activation of the above mentioned targets.

NF-κB signaling is also a requirement in cellular growth through the regulation of cyclins^25^. Cyclin D1 is responsible for regulating the G1/S phase transition of the cell cycle. In the presence of appropriate stimulus, cyclin D1 is induced and dimerizes with CDK4 or 6 to phosphorylate retinal blastoma (Rb) protein, which suppresses E2F transcriptional activity for S-phase entry. In this study, cyclin D1 expression increased at 24 h when stimulated with DPP and the expression was attenuated in the presence of p65 inhibitors TPCA-1 and JSH-23 confirming the specificity of NF-κB in regulating cyclin D1. Results also demonstrated that NF-κB is required for cell proliferation. Significant upregulation of cyclin B1, which promotes cell proliferation, were observed from 4 to 24 h and repressed by blocking p65 signaling. Upregulation of cyclin A1 was also observed with NF-κB activation. Cyclin A is involved in both S phase and the G2/M transition of the cell cycle through its association with distinct cdks. Upregulation of cyclin E1 at 4 and 16 h indicate cell –cycle progression and DNA synthesis with mitosis. Thus, NF-κB signaling activated by DPP, functions to regulate both cell growth and differentiation.

Overall, we demonstrate that DPP activates NF-κB signaling in dental pulp stem cells. Specifically the p65 subunit of NF-κB is responsible for the odontogenic differentiation of DPSCs. These changes are associated with elevated expression of early differentiation markers such as Runx2, osterix, alkaline phosphatase and FGF2. Inhibition of IKKβ by specific small molecules suppressed both cell proliferation and the terminal differentiation of the DPSCs into odontogenic lineage. In sum, these data indicate a critical signaling function for DPP in the differentiation of DPSCs by modulating the activity of p65 and IKKβ.

## Materials and methods

### Cell culture

Dental pulp stem cells (DPSCs, a kind gift from Dr. Shi, University of Pennsylvania) were cultured in growth media [α-minimum Eagle's medium (Thermo Fisher Scientific) supplemented with 10% fetal bovine serum (FBS; Thermo Fisher Scientific) and 1% Antibiotic–Antimycotic (100 ×) (Thermo Fisher Scientific)] in a humidified CO_2_ incubator at 37 °C. Cells were split at 1:3 ratio at ~ 90% confluency (passage), and cells from passage 6 to 12 were used in all experiments.

### Immunoblotting

Recombinant DPP protein was expressed and purified as described previously^9^. DPSC cells were seeded at 70% confluency in growth media. Before stimulation the cells were placed in growth media with reduced serum (1% FBS) overnight. Cells were stimulated with DPP (500 ng/ml) before or after treatment with small molecule inhibitors.

Following NF-kB pathway inhibitors were used: 10 µM TPCA-1 (2 carbamoylamino)-5-(4-fluorophenyl) thiophene-3-carboxamide), a potent IKK-2 inhibitor^27^, 10 µM JSH-23 (4-Methyl-*N*1-(3-phenyl-propyl)-benzene-1,2-diamine) an inhibitor specifically reduce p65 and DNA complex formation^16,39^ is a cell-permeable, selective inhibitor of nuclear translocation of NF-κB p65 and its transcription.

After 1 h incubation, cells were harvested and lysed in RIPA buffer (Cell signaling) containing protease and phosphatase inhibitor cocktail (Millipore). Centrifugation was then performed at 14,200 rpm for 15 min at 4 °C and the supernatants were used as total cellular proteins. Proteins concentrations were measured with Bio-Rad Protein Assay Dye Reagent (BIO-RAD,) with BSA as standard. 30 μg of total proteins were separated by electrophoresis on a 10% SDS-PAGE gel and transferred onto a polyvinylidene fluoride (PVDF) membrane (BIO-RAD). The membranes were incubated with the following primary antibodies: anti-NF-kappaB p65 rabbit polyclonal antibody (abcam), anti-phospho-NF-kappaB p65 rabbit monoclonal antibody (Cell Signaling), overnight at 4 °C. They were washed with PBS, incubated with HRP conjugated anti-mouse or anti-rabbit secondary antibodies (Cell signaling). The bands were visualized using SuperSignal™ West Pico chemiluminescent Substrate (Thermo Scientific) with X-ray films according to the manufacturer’s protocol. The membrane were then stripped with Restore PLUS Western Blot Stripping Buffer (Thermo-Scientific) and probed with anti-α-Tubulin mouse monoclonal antibody (Sigma) as internal control. The images were scanned and band densities were measured using ImageJ (1.52c).

### Immunofluorescence

DPSCs were seeded on glass coverslips in growth media at 70–80% confluence. Cells were cultured at reduced serum concentration (1%) overnight. Cells were treated with 10 µM TPCA-1 for 30 min before the addition of DPP (500 ng/ml). At the indicated time points, DPSC cells were fixed in 10% neutral formalin at 4 °C for 1 h. The cells were permeabilized with 0.25% Triton-X in PBS for 30 min, and were incubated with an anti-NF-kappaB p65 rabbit polyclonal antibody (abcam) overnight at 4 °C, followed by incubation with fluorescent goat-anti Rabbit Alexa594 (Abcam) at RT for 1 h. The cover glass was mounted onto a glass slide using mounting agent with DAPI (Vectorlab) and cells were imaged with a Zeiss 710 Meta Confocal Microscope at the UIC RRC Facility.

### Quantitative real time PCR (qRT-PCR)

DPSC cells were treated as described above and harvested at indicated times points. The total RNA were obtained from cells using TRIzol Reagent (Invitrogen). cDNAs were synthesized and real-time PCR was conducted as described previously^3^. The gene expression levels were estimated by the 2^−ΔΔCT^ method with GAPDH gene expression level as a internal control. Primers were synthesized by IDT (Integrated DNA Technologies, Inc.). The primer sequences are listed in Table S1.

### Transient transfection and luciferase assay

One day prior to transfection, 3 × 10^5^ cells were seeded into one well of a 12-well tissue-culture plate (BD Biosciences, San Jose, CA). After 12 h, co-transfections were conducted using the transfection vector mixture (total 333 ng) containing NF-κB RE luc reporter construct (300 ng, a kind gift from Dr. Lyndon F Cooper) or PGL Basic reporter construct (Promega). CMV/Renilla luciferase vector (33 ng, Promega) was used as transfection efficiency control. 0.6 μl of transfection agent Lipofectamine 2000 (Invitrogen) was added to the mixture, and co-transfections were conducted according to the manufacturer's instruction. Sixteen hours later, the cells were treated with TPCA-1 at 10 µM for 30 min followed by addition of DPP (500 ng/ml). After 48 h, cell lysates were prepared using passive lysis buffer (Promega). Luciferase activities in the lysates were measured using a dual luciferase assay system (Promega) with a plate reader (Synergy 2, BIOTEK).

### Chromatin immunoprecipitation

Bioinformatics analysis was used to identify the NF-κB p65 binding consensus sequence GGGNNNNNCC in the promoter regions of NF-κB target genes. To test the possibility that DPP activates gene expression by binding of NF-κB p65 subunit to the putative promoter elements of genes involved in odontogenic differentiation, specific primer pairs flanking each binding element were designed and used to detect the DNA fragments by RT-PCR. Two pairs of primers were synthesized for hOsx_65A + B and hTWIST1_A + B which have two binding sites in close proximity. Primers amplifying random regions within or adjacent to the NF kB promoter binding element were synthesized and designated as negative controls. Chromatin immunoprecipitation (ChIP) assays were performed with a commercially available CHIP-IT high sensitivity kit following the manufacturer's protocol (Active Motif North America, Carlbad, CA). Briefly, 1 × 10^6^ DPSC’s were treated with DPP (500 ng/ml) for 1 or 2 h and anti-p65 antibody (ab16502, Abcam) was used in immunoprecipitation, while the normal isotype-matched IgG from the same species served as negative control. RT-PCR was used to amplify DNA fragments using specific primers. Relative amount of the DNA fragments were calculated as percentages to that of the input DNA that were used in the ChIP assay. The primer sequences are listed in Table S2.

### Cell proliferation

DPSC cells were seeded in 96 well plate and cultured for 24 h. They were treated with 10 µM TPCA-1 for 30 min followed by the addition of DPP at 500 µl/ml (0 h). Cells were incubated at 37 °C, 5% CO_2_, for the duration as indicated, and fresh media with TPCA-1 and DPP as required were added at 48 h. CellTiter 96 Aqueous One Solution Reagent (Promega, Cat. No. G3580) was used to monitor the cell numbers according to the manufacturer’s instructions. The absorbance was recorded on a multiplate reader at 490 nm.

To measure cyclin D1 (CCND1) protein expression levels, DPSCs were incubated with αMEM-1% FBS overnight, pretreated with TPCP-1 for 30 min and then stimulated with DPP (500 ng/ml). At the indicated time points, the whole cell lysates were extracted and Western blotting performed with CCND1 antibody (Cell signaling). RT-PCR was performed to assay genes for cell-cycle progression. For this, DPSCs were pretreated with TPCA-1 or JSH-23 for 30 min and then stimulated with DPP (500 ng/ml).

### In vitro mineralization assay and nodule detection by alizarin red staining

DPSC’s were seeded in growth media until 80% confluent. Then they were cultured in osteogenic differentiation media [growth medium containing 10 mM β-glycerophosphate (Thermo Fisher Scientific), 0.50 mM ascorbic acid (Sigma-Aldrich), and 10 nM dexamethasone (Sigma-Aldrich)] with or without DPP (500 ng/ml) and p65 inhibitor TPCA-1 for 0, 7, 14 and 21 days. At each time point, the cells were washed with PBS and fixed in 10% neutral formalin at 4 °C for 4 h. The cells were stained with 2% Alizarin Red S Solution (Sigma) for 30 min, then rinsed with water. The plates were scanned to visualize the overall staining pattern and high magnification images were obtained with a light microscope. Colorimetric method was used as published for quantitative analysis of calcium present in the cell culture matrix^3,24^.

### Immunohistochemical analysis

DSPP null mice and their matched wild type mice were bred and head sections collected and used for IHC (immunohistochemical analysis). All mouse care and experimental procedures were approved and conducted in strict accordance with the guidelines and regulations stipulated by the Institutional Animal Care and Use committee at the University of Illinois at Chicago and in compliance with the ARRIVE (Animal Research: Reporting of in Vivo Experiments) guidelines. Formalin fixed paraffin-embedded specimen and paraffin block sectioning were conducted as described previously^24^. Primary antibodies used were: OCN (ab93876), OSX (sc-393325), PTX3 (sc-373951), and p65 (abcam16502). Sections were developed with VECTASTAIN^®^ ABC HRP kit (Vector Laboratories,) or (M.O.M.™) Elite Peroxidase Kit (Vector Laboratories) as per manufacturer’s instruction. The DAB Peroxidase (HRP) Substrate Kit (Vector Laboratories) was used for antigen detection. Sections were imaged with Carl Zeiss Axio Observer D1 inverted microscope. ImageJ (1.50c) and IHC Profiler plugin was used to quantitate positive staining within area of interests (AOI) and represented as percentages. Data extracted from at least 6 AOIs per sample were used to obtain statistical significance.

### Statistical analysis

Data were presented as the mean ± standard deviation of at least 3 independent experiments. Statistical significance of differences were calculated using the Student’s t test. Significance of p ≤ 0.05 was considered significant.

## Supplementary Information

Supplementary Information.
