# Supplementary material for: DPP promotes odontogenic differentiation of DPSCs through NF-κB signaling
Source: Sci Rep. 2021 Nov 11;11:22076. doi: 10.1038/s41598-021-01359-3 (PMC8586344; doi:10.1038/s41598-021-01359-3)

SUPPLEMENTARY INFORMATION

DPP promotes odontogenic differentiation of DPSCs through NF-κB signaling: Implications in tissue regeneration

Yinghua Chen, Adrienn Petho, Amudha Ganapathy and Anne George*

Brodie Tooth Development Genetics & Regenerative Medicine Research Laboratory,

Department of Oral Biology, University of Illinois at Chicago,

Chicago, Il 60612, USA

* To whom correspondence should be addressed. Tel.: 312-413-0738; Fax: 312-996-6044; E-mail: anneg@uic.edu

Table S1.DNA oligoes for quantitative RT-PCR of gene expression

Gene Name	Accession	Sequence
ALP	NM_001127501	ACTGGTACTCAGACAACGAGAT
		ACGTCAATGTCCCTGATGTTATG
BMP4	NM_001202	AAAGTCGCCGAGATTCAGGG
		GACGGCACTCTTGCTAGGC
FGF2	NM_002006	AGTGTGTGCTAACCGTTACCT
		ACTGCCCAGTTCGTTTCAGTG
GAPDH	NM_001357943.2	GGAGCGAGATCCCTCCAAAAT
		GGCTGTTGTCATACTTCTCATGG
MMP1	NM_002421.3	GATGTGGAGTGCCTGATGTG
		CTGCTTGACCCTCAGAGACC
MMP3	NM_002422	TGAAGACTTTCCAGGGATTGAC
		GGCAGGAGAAAACGAACATTTC
OCN	NM_199173	GGCGCTACCTGTATCAATGG
		GTGGTCAGCCAACTCGTCA
OPG	NM_002546.4	CAAAGTAAACGCAGAGAGTGTAGA
		GAAGGTGAGGTTAGCATGTCC
OSX	NM_001300837.2	AGCAGGCACAAAGAAGCCGT
		TGCCTGCAGGTGAAAGGAGC
PTX3	NM_002852	CATCTCCTTGCGATTCTGTTTTG
		CCATTCCGAGTGCTCCTGA
RANKL	NM_033012.4	CGACATCCCATCTGGTTCC
		GCTGGTTTTAGTGACGTACACC
Runx2	NM_001015051	TGGTTACTGTCATGGCGGGTA
		ACTGCCCAGTTCGTTTCAGTG
TWIST1	NM_000474.4	GGAGTCCGCAGTCTTACGAG
		TCTGGAGGACCTGGTAGAGG

Table S2. DNA oligoes for quantitative PCR of NF kB binding elements

DNA site ID	Sequence
ALP A	AATGGGTGCCTCCAATTCCCTC
	CTTGCTCACAAATGCCAAGG
ALP B	CCTTTACGTCTCTAAAGAGAG
	GTCTCTGTCTTTATCCGTCTC
ALP C	CAAGAGACGCAGAAGGAAGATG
	ACTCTCGGTCTCTCTGCCTC
ALP D	CGTTCCTAGCTCCGCTCCCGGC
	ACAAGCGCGTGGGAGCACGGA
ALP random	ATGATCTCTCTGGGCCTCAG
	GCCCAGAGAACCAATTTTAGCC
BMP4_A	GTAGCAGCATAGGAGCAATAG
	CAGAGTTCGTGAGAAGAATCAG
BMP4 random	GAGTGGACATTGGAGATTAAGC
	TATCTCTGCTGGGTCCAAGT
MMP1 A	GAGTAAGATATCAGTCTTGACGCAG
	CAGTGGAGAAACACTGGCCTG
MMP1_B	GACTAGGACTACAGGTGCATG
	CATGGTGGCTCAAGCCTATAATC
MMP1 C	AGTCAGACAGCCTCTGGCTTTC
	CAGAACAGCAGCAGCAGCAGT
MMP1 random	CCTAGCACCAAGGAGCGAAGA
	TGTACCGGATGATGAAAAGGCTGG
OCN A	GTGAGTGAGGAGACAGGTTAG
	CTCCCGTAGTGCTCCGATAAG
OCN B	GAGCTCAGCCAGTGCTCAAC
	AGGATATTGTGGTTGGGAGCTGC
OCN random	GTGTGCCTGTAGTCCCAGCTAC
	GTGAAGTGGAAGAAGCAAGAG
hOsx A+B	GATAATGGAGGTTAATGGGGCG
	GCCGTTCCAAGCGAAGAAGAG
Osx random	CGACAGAGCGAGACTCCGTCT
	AGGCTGGTGTCGAACTCCTGAC
PTX3_A	GTGGTTTGCTTCCTCATATGTTC
	CTAGCACAAAGAGGTAGGAAGC
PTX3 B	CTTTCCTCTCTATCTTTGGCTC
	GGTTTCGAGGCAAGGATCATG

PTX3 C	GTGCAACTTCCACATTTCCCTC
	GAGTAATGCTGGTGGCACTGC
PTX3_random	CGTCCTCCTGAACAATGAAATG
	GCTCAAGGCATGGATTAAGTG
TWIST1 A+B	GCTCACGAAGCCTAATTGTC
	CTAACAGCTGGCAATGCCAAC
TWIST1 C	CATTGCTGCTGTCACAGCCACTC
	GGTCTAACAATTCGTCCTCCC A
TWIST1_random	CAGAATTTCTGCCGCATTCTC
	TGTTTAGTGAGCCGCTGCCT

Supplementary Figure 1

Anti-rabbit secondary antibody control used to demonstrate the specificity of primary antibody binding in Fig 9.

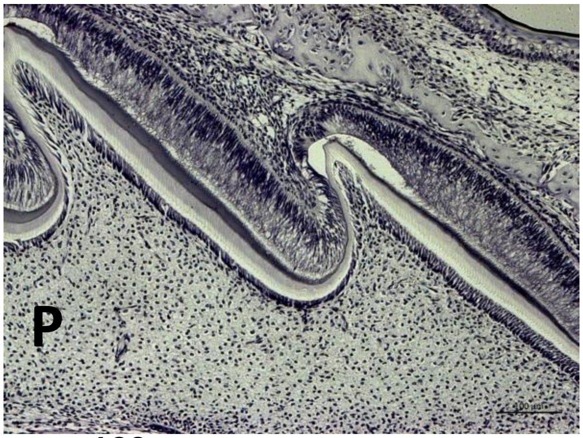

: 100 μm

Supplementary Figure 2

Full length blots that were cropped and presented in figure 1.

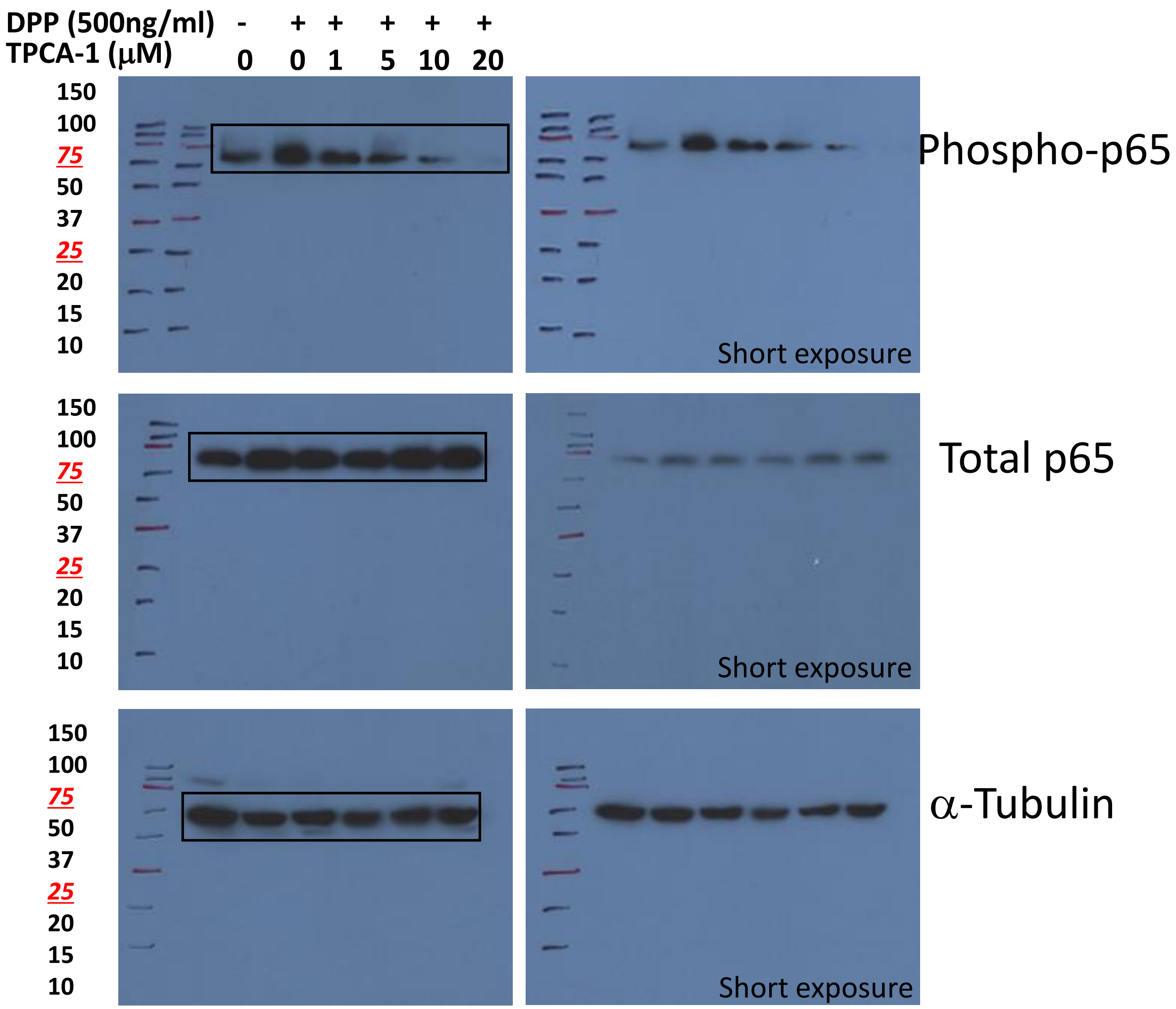

Supplementary Figure 3

Full length blot images that were cropped and presented in figure 3F.

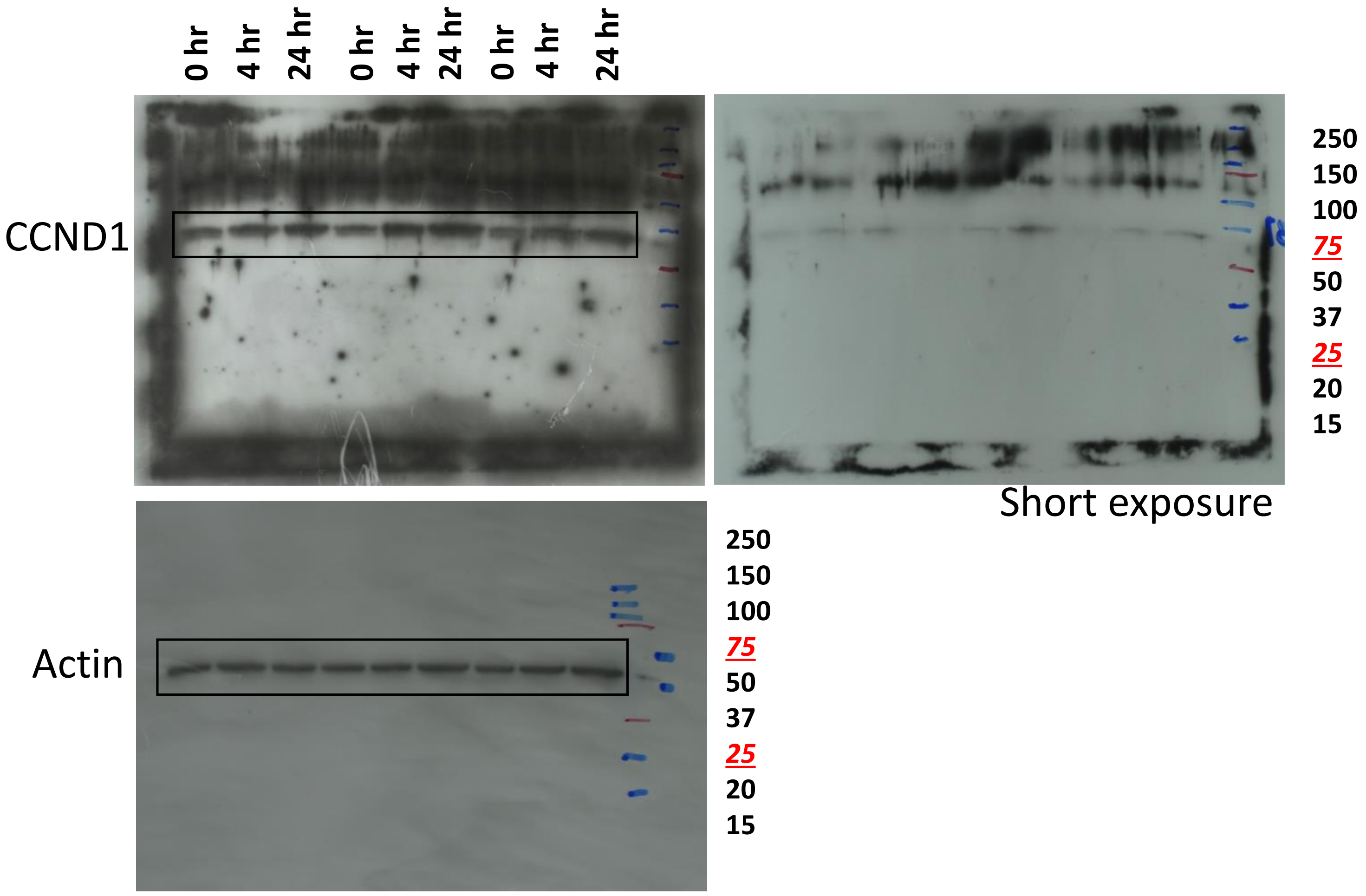

Supplement: Supplementary file 1 — Supplementary Information. [file 41598_2021_1359_MOESM1_ESM.pdf]
